# Focus on a 2019-novel coronavirus (SARS-CoV-2)

**DOI:** 10.2217/fmb-2020-0063

**Published:** 2020-06-11

**Authors:** Ling-Pu Zhang, Meixian Wang, Yanping Wang, Jun Zhu, Nannan Zhang

**Affiliations:** ^1^National Center for Birth Defect Monitoring, Key Laboratory of Birth Defects & Related Diseases of Women & Children, Ministry of Education, West China Second University Hospital, & State Key Laboratory of Biotherapy, Sichuan University, Chengdu, PR China; ^2^Energy Saving Technology Service Center (Chengdu Energy Conservation Supervision Center) of Chengdu, Sichuan University, Chengdu, PR China

**Keywords:** clinical characteristics, epidemiology, genomic, laboratory testing, SARS-CoV-2, therapeutics

## Abstract

A new coronavirus, severe acute respiratory syndrome coronavirus 2, was first discovered in Wuhan, China, in December 2019. As of April 7, 2020, the new coronavirus has spread quickly to 184 countries and aroused the attention of the entire world. No targeted drugs have yet been available for intervention and treatment of this virus. The sharing of academic information is crucial to risk assessment and control activities in outbreak countries. In this review, we summarize the epidemiological, genetic and clinical characteristics of the virus as well as laboratory testing and treatments to understand the nature of the virus. We hope this review will be helpful to prevent viral infections in outbreak countries and regions.

The 2019-novel coronavirus, which was first reported in Wuhan, Hubei Province, China, was classified as severe acute respiratory syndrome coronavirus 2 (SARS-CoV-2) by the International Committee on Taxonomy of Viruses [1]. Infection has spread to approximately 184 regions and countries including Japan, South Korea and the USA, Africa, Europe and Australia [2]. As of April 7, the total number of infections on the Chinese mainland was 83,071 (Figure 3A), the total number of recovered patients was 77,468 (Figure 3B) and the number of deaths had increased to 3340 (Figure 3C). SARS-CoV-2 belongs to the *Betacoronavirus* lineage, subgenus *Sarbecovirus* [3,4]. Symptoms of this virus include fever, cough, shortness of breath, leukopenia and pneumonia in both lungs [5]. Patients of advanced age (>50 years) exhibit significant comorbidities [6]. Originally, it was thought to be mainly associated with the elderly, but now the virus is affecting younger people: even children [7]. Patients with severe viral infections need intensive care and are at high risk of death [5]. However, except among elderly cases and those with chronic disease, the mortality rate of COVID-19 appears to be low at this point [8].

SARS-CoV-2 is one of seven coronaviruses that have been identified thus far [9]. The other coronaviruses are 229E, HKU1, OC43, NL63, severe acute respiratory syndrome (SARS) and Middle East respiratory syndrome (MERS)-CoV [9–12]. Of these, 229E, HKU1, OC43 and NL63 have relatively low pathogenicity [10,13,14]. SARS-CoV and MERS-CoV can cause fatal pneumonia with death rates of 10 and 37%, respectively [11,15]. SARS-CoV-2 is associated with person-to-person transmission and with a low mortality rate (2–3%) [6]. As of 7 April 2020, COVID-19 has spread to 184 countries (Figure 3D), infected at least 1,347,804 patients worldwide (Figure 3D & G), and has been the cause of 74,596 deaths globally (Figure 3F & I) (data from Johns Hopkins resource center). Currently, specific vaccines and medicine for SARS-CoV-2 infection are being developed [16]. This paper summarizes the epidemiological, genetic, clinical characteristics, laboratory diagnosis, animal models and therapeutics of this virus, which could be critical for the prevention of SARS-CoV-2.

## Discovery & source of SARS-CoV-2

On 12 December 2019, a new type of coronavirus was identified in Wuhan Hubei Province in China [17]. The full genome of the novel coronavirus was posted in GenBank and the Global Initiative on Sharing All Influenza Data by Chinese health authorities and the Centers for Disease Control and Prevention (CDC) of America [8]. It was named severe acute respiratory syndrome coronavirus 2 (SARS-CoV-2) by the International Committee on Taxonomy of Viruses on 11 February 2020 [1]. The disease caused by SARS-CoV-2 was named COVID-19 by the World Health Organization (WHO). On 30 January 2020, the WHO declared the outbreak of COVID-19 to be a global health emergency and further labeled it a pandemic on 11 March 2020.

## Spread of the virus

SARS-CoV-2 was first reported to be from the South China seafood market [17]. However, subsequent studies have shown that more than 45% of the early patients before 1 January 2020 were not linked with this market [18]. SARS-CoV2 and SARS-CoV are closely related to each other and originated in bats, which most likely serve as reservoir host for these two viruses. MERS-CoV is also considered to originate from bats; however, the reservoir host is unequivocally dromedary camels [19] (Table 1). Bats and minks may be intermediate hosts of SARS-CoV-2 because SARS-CoV-2 shares 96.2% homology with bat coronavirus (bat CoV RaTG13) in Yunnan Province [8,20]. Due to their use in medicine and food, bats have been regarded as the ultimate host for transmission to humans [21]. Animals such as the snake and bamboo rat have also been considered as intermediate hosts of SARS-CoV-2. Snakes are reportedly the most likely wildlife animal reservoir of the virus because their relative synonymous codon usage bias was close to that of the SARS-CoV-2 virus [21]. However, virologists have stated that no evidence supports this hypothesis for snakes as a SARS-CoV-2 host. The bamboo rat was also predicted to be an intermediate host of SARS-CoV-2 [22]. Scientists are currently working to identify the source of SARS-CoV-2, including possible intermediate animal vectors.

**Table 1.** Comparison between severe acute respiratory syndrome coronavirus 2, severe acute respiratory syndrome coronavirus and Middle East respiratory syndrome coronavirus.

Virus	SARS-CoV-2	SARS-CoV	MERS-CoV	Ref.
Outbreak year	2019	2003	2012	
Outbreak countries	More than 184 countries, including USA, China, Japan, Korea, Italy, etc.	29 countries, including China, Vietnam, Singapore and Canada	>27 countries, mainly in Saudi Arabia, South Korea, Jordan and Qatar	[81]
Natural reservoir	Not identified	Bat	Bat	[81]
Receptor	ACE2	ACE2, CD206	Dpp4 (CD26)	
Fatality rate%	Not identified, at least 2–3%	10%	37%	[51,83]
Basic reproductive number	1.4–6.4	2–5	<1	[51]
Median incubation time	5.2 days	5 days	5 days	[50]
Clinical symptoms	Fever (98%), sough (77%), dyspnea (63.5%), myalgia (11.5%), malaise (35%) and so on	Fever (>99%), cough (62%–100%),chills or rigor (15%–73%), diarrhea 20%, dyspnea (40%)	Fever (77%), cough (90%), dyspnea (68%), sputum production (40%), odynophagia (39%), digestive system /signs (20%), hemoptysis (4.3%), myalgia (43%) and headache (20%)	[53,69,85]
Radiology	Critically ill patients with bilateral multiple lobular and subsegmental areas of consolidation; mild patients with bilateral ground-glass opacity and subsegmental areas of consolidation almost 100% patients with abnormal CT	Unilateral/bilateral ground-glass opacities or focal unilateral/bilateral consolidation. Chest radiography or CT abnormal rate was >94%	Unilateral/bilateral patchy densities or infiltrates, bilateral hilar infiltration, segmented/lobar opacities, ground-glass opacities and possible small pleural effusions. Chest radiography or CT abnormal rate was between 90% to 100%	[50]
Cytokines	IL-1β, IL1RA, IL-7, IL-8, IL-9, IL-10, basic FGF, GCSF, GMCSF, IFN-γ, IP10, MCP1, MIP1α, MIP1β, PDGF, TNF-α and VEGF increased, ICU patients also had higher GCSF, TNF-α and T-helper-2 (Th2) cytokines (e.g., IL-4 and IL-10) also increased	IL-1β, IL-6, IL-12, IFN-γ, IP10 and MCP-1 increased	Increased concentrations of proinflammatory cytokines (IFN-γ, TNF-α, IL-15 and IL-17)	[6,50]
Treatment medicine	Corticosteroids, remdesivir, combination of lopinavir and ritonavir, type I interferon and so on	Lopinavir and ritonavir, corticosteroids, IFN-α, IVIG	IFN-β, lopinavir and ritonavir, mycophenolic acid	[67,83]

ACE2: Angiotensin-converting enzyme 2; CT: Computed tomography; DPP4: Dipeptidyl peptidase IV; FGF: Fibroblast growth factor; GCSF: Granulocyte colony stimulating factor; GMCSF: Granulocyte/monocyte colony stimulating factor; ICU: Intensive care unit; IFN-α: Type 1 interferon; IFN-β: Interferon beta; IFN-γ: Interferon gama; IL-1β: Interleukin 1 beta; IL1RA: Interleukin-1 receptor antagonist; IL-6: Interleukin 6; IL-7: Interleukin 7; IL-8: Interleukin 8; IL-9: Interleukin 9; IL-10: Interleukin 10; IL-15: Interleukin 15; IL-17: Interleukin 17; IVIG: Intravenous immunoglobulin; MERS-CoV: Middle East respiratory syndrome coronavirus; TNF-α: tumor necrosis factor-alpha; VEGF: Vascularendothelial growth factor; SARS-CoV-2: Severe acute respiratory syndrome coronavirus 2.

The virus can be transmitted not only from animals to humans but also from humans to humans [23]. A *Lancet* report demonstrated that the virus could have recently acquired the ability to transmit between humans [6]. A report of five patients in a family cluster who traveled to Wuhan and were infected with SARS-CoV-2 was the first report directly illustrating that the virus is capable of person-to-person transmission in hospital and family settings [23].

SARS-CoV-2 can spread via direct contact and respiratory droplets. Respiratory particles are spread while breathing, speaking, coughing or sneezing [24]. In addition, aerosol and fomite transfer may promote transmission of the virus according to a study in the New England Journal of Medicine [25]. Aerosolized virus may be generated by respiratory and surgical procedures. The study showed that the half-life of the virus is about 1.1–1.2 h, and it remained viable for 3 h in aerosols. Meanwhile, the virus on plastic, stainless steel, copper and cardboard remained stable for 4–72 h [25]. These results indicate that aerosol or fomites may be able to spread SARS-CoV-2 [25]. A fluid-resistant (Type-R) surgical face mask is used to protect against droplets.

Fecal-oral transmission may also play an important role in SARS-CoV-2 spread [26]. Xiao and colleagues showed that 53.42% of 73 hospitalized COVID-19 patients had SARS-CoV-2 RNA in stool specimens, and the duration time of positive stool results ranged from 1 to 12 days [27]. In addition, viral nucleic acid in 64.29% patients remained positive in the feces after SARS-CoV-2 RNA in pharyngeal swabs turned negative. Furthermore, positive SARS-CoV-2 RNA in stool specimens was not associated with gastrointestinal symptoms [28]. Together, these findings indicate the possibility of SARS-CoV-2 via the fecal-oral transmission.

The basic reproduction number (R0) of this virus reflects the dynamics of transmission during this coronavirus outbreak. The WHO has estimated that SARS-CoV-2 has a reproduction number of 1.4–2.5. However, a recent study shows the average R0 to be 3.28 (median: 2.79; interquartile range [IQR]: 1.16), an R0 considerably higher than the WHO estimate at 1.95 [29]. In an earlier phase of the outbreak, Li *et al.* reported that the mean incubation period of the virus is 5.2 days (95% CI: 4.1–7.0), the epidemic doubled in size every 7.4 days, and the R0 was estimated to be 2.2 (95% CI: 1.4–3.9) [30]. Zhao and collaborators estimated an R0 ranged from 2.24 (95% CI: 2.49–2.63) to 3.58 (95% CI: 2.89–4.39) [31]. In another study, R0 was computed to oscillate between 3.30 (95% CI: 2.73–3.96) and 5.47 (95% CI: 4.16–7.10) [32]. In addition, the study reported a transmission rate within Wuhan of 1.94 days (95% CI: 1.25–6.71), an infectious period of 1.61 days (95% CI: 0.35–3.23) and an R0 value of 3.11 (95% CI: 2.39–4.31) [32]. Based on the nowcasting and forecasting approach, Wu *et al.* showed an estimated reproduction number of 2.68 (95% CI: 2.47–2.86), and an epidemic doubling time of 6.4 days [33]. Recently, Tang *et al.* calculated the R0 as 6.47 (95% CI: 5.71–7.23), using Mathematical SEIR-type epidemiological model [34]. Nevertheless, SARS-CoV-2 has demonstrated a higher transmission rate than that of SARS-CoV and MERS-CoV. Variation in viral transmissibility should be considered, and estimates of the reproduction number may change in the future.

## Genomic characterization & structure of SARS-CoV-2

**Figure 1.**
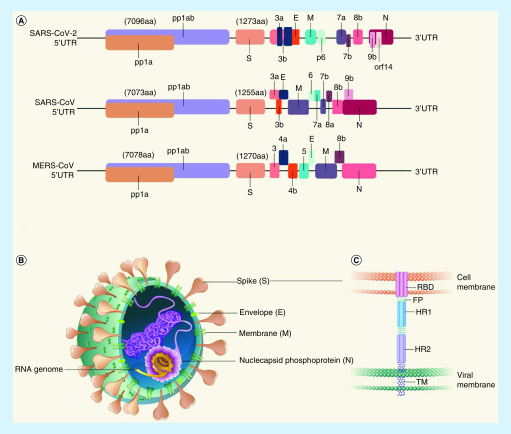
Genome composition, schematic diagram and functional domains of spike protein in SARS-CoV-2. **(A)** Schematic diagram of the genome organization and the encoded proteins of SARS-CoV-2, SARS-CoV and MERS-CoV. Structural proteins included spike (S), envelope (E), membrane (M) and nucleocapsid (N). Pp1a and pp1ab were all encoded by the *orf1a* or *orf1ab* genes. Pp1a protein contained ten nonstructural proteins (nsps) (nsp1-nsp10). Pp1ab protein contains 15nsps (nsp1-nsp10 and nsp12-nsp16). The protein-encoding genes of the genome of SARS-CoV-2 were from GeneMarkS or ORFfinder. The differences in the arrangement of the envelope (E), membrane (M) and nucleoprotein (N) among SARS-CoV-2, SARS-CoV and MERS-CoV are shown at 3′ end. **(B)** Schematic diggram of SARS-CoV-2 virus. SARS-CoV-2 was an RNA virus. The envelope was a lipid bilayer membrane. Matrix protein, below the lipid membrane, formed a shell which could give strength and rigidity to the lipid membrane. RNA segments were in the interior of virus, which were the genetic materials of the virus. Spike protein was the structural protein responsible for the crown-like shape of the coronavirus particles. S-protein was processed at the S1/S2 cleavage site by host cell proteases, during infection. **(C)** The representative scheme of functional domains in S protein of SARS-CoV-2. FP: Fusion peptide; HR1: Heptad repeat 1; HR2: Heptad repeat 2; RBD: Receptor-binding domain; TM: Transmembrane domain.

*SARS-CoV-2* is similar to *SARS-CoV*. Both single-stranded RNA viruses share 82% nucleotide identity, and *SARS-CoV-2* shares 89% identity with *SARS-like CoVZXC21* [35]. The genome of *SARS-CoV-2* has 29,891 nucleotides encoding 9,860 amino acids. Genetically, *SARS-CoV-2* is similar to *SARS-CoV* (about 79%) and *MERS-CoV* (about 50%) [36]. The virus contains a replicase, spike (S) protein, envelope (E) protein, membrane (M) protein and nucleocapsid [35] (Figure 1A & B). However, *SARS-CoV-2* lacks the hemagglutinin-esterase gene, which is found in lineage A β-COVs.

*SARS-CoV-2* has 12 open reading frames (ORFs) encoded by nine subgenomic mRNAs that carry nine transcription regulatory sequences, two terminal untranslated regions (UTR) and a conserved leader sequence [37]. The large replicase polyprotein pp1a contains ten nonstructural proteins (nsp1–nsp10), and pp1ab contains 15 nonstructural proteins (nsp1–nsp10, nsp12–nsp16), which are all encoded by *ORF1a* and *ORF1ab* [38] (Figure 1A). With the exception of nsp3 and nsp5, which are cysteine proteases, most nonstructural proteins play an important role in the transcription and replication of SARS-CoV-2 [39]. *Pp1ab* has different lengths in COVID-19, SARS-CoV and MERS-CoV of 29,844 bp (7096 aa), 29,751 bp (7073 aa) and 30,119 bp (7078 aa), respectively (Figure 1A). Furthermore, there is no obvious difference between SARS-CoV-2and SARS-CoV nonstructural proteins and ORFs.

The spike glycoprotein plays an important role in binding to receptors on host cells and, therefore, is involved in host tropism [40]. SARS-CoV-2, SARS-CoV and MERS-CoV have S proteins containing 1,273, 1,255 and 1,270 aa, respectively (Figure 1A) [36]. The S protein mediates entrance into human respiratory epithelial cells by interacting with the cell-surface receptor angiotensin-converting enzyme 2 (ACE2) [35,41]. It is comprised of S1 and S2 subunits (Figure 1C), and the S1 subunit shares approximately 70% identity with that of human SARS-CoV and bat SARS-like CoVs (SL-COVZXC21 and ZC45). The S1 subunit has an N-terminal domain and a receptor-binding domain (RBD) that are both responsible for the binding of virions to host cells [16]. Both SARS-CoV-2 and SARS-CoV bind to ACE2 through the C-terminal domains (CTD) of their S1 subunits, and MERS-CoV utilizes the CTD to bind proteinaceous dipeptidyl peptidase 4 (DPP4) [42]. The RBD of SARS-CoV-2 has 73% identity to that of SARS-CoV [43]. The transmissibility of the SARS-CoV-2 virus is greater than that of SARS-CoV, which may be because the RBD of SARS-CoV-2 is slightly different from that of SARS-CoV. The S2 subunit shares 99% identity with two bat SARS-like CoVs and human SARS-CoVs [38]. The S2 subunit contains a fusion peptide (FP) and heptad repeats (HRs) 1 and 2 (Figure 1C). After the S1 RBD binds to the ACE receptor on the host cell, the FP of S2 is inserted into the host cell membrane, and then HR1 and HR2 form a six-helix bundle (6-HB), which helps the virus fuse with host cell membranes [16,44]. SARS-CoV-2 envelope (E) protein, matrix protein, accessory proteins p6 and p8, nonstructural protein 7 (nsp7), and nsp13 are homologous with those of SARS virus [39].

Therefore, the SARS-CoV-2 virus has a high level of identity with SARS-CoV. This suggests that an anti-SARS-CoV antibody, which could cross-react with the SARS-CoV-2 S protein, may be useful to treat patients with the virus.

## Clinical characteristics of patients infected with SARS-CoV-2

SARS-CoV-2 infection has caused clusters of severe respiratory illness similar to that of SARS-CoV. The virus causes symptoms such as fever, cough, shortness of breath, leukopenia and pneumonia in both lungs [6]. The symptoms are observed approximately 5.2 days after the SARS-CoV-2 infection [5]. In a study published in *The Lancet*, 41 of 41 patients who were identified as positive for SARS-CoV-2 infection presented with pneumonia and abnormal chest computed tomography (CT) [6]. COVID-19 symptoms included fever (98%), cough (76%) and myalgia or fatigue (44%). Less common symptoms such as sputum production (28%), headache (8%), hemoptysis (5%) and diarrhea (3%), were also observed [6].

**Figure 2.**
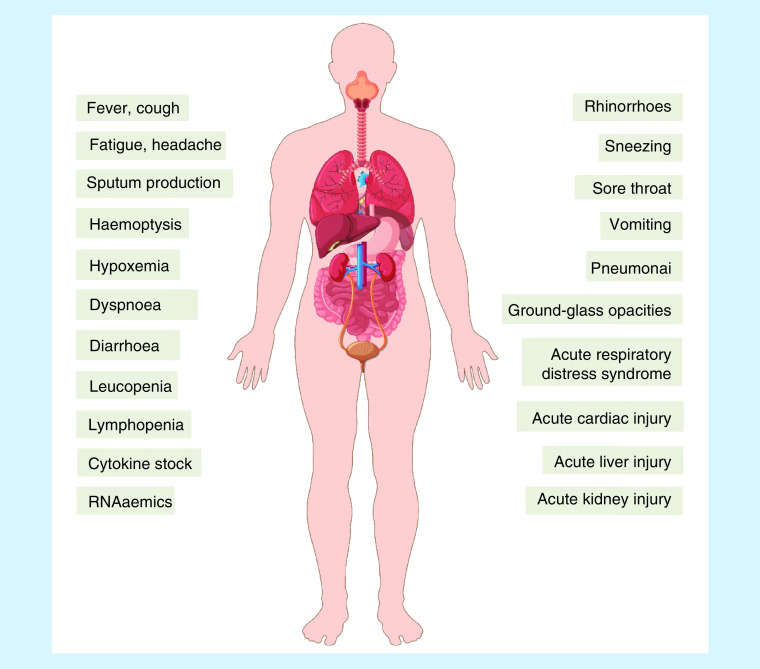
Clinical disorders caused by COVID-19 infection. Patients infected with COVID-19 had some unique clinical features including rhinorrhoea, sneezing, sore throat. Most patients had haemoptysis dyspnea, fever, headache, fatigue, sputum production, pneumonia and ground-glass opacities. However, only a low percentage of patients developed intestinal symptoms such as diarrhea and vomiting. Leucopenia, lymphopenia, pro-inflammation cytokines increasing, acute respiratory distress syndrome and acute organs damages (such as cardiac, liver or kidney) were common features in some intensive care unit patients.

Another clinical study containing 138 patients showed that common symptoms were fever, fatigue, dry cough, lymphopenia, prolonged prothrombin time and an increased lactate dehydrogenase level (Table 1 & Figure 2) [5]. Common complications included shock, acute respiratory distress syndrome, acute renal injury, acute liver failure, arrhythmia, RNAaemia and acute cardiac injury [45]. In addition, it is now understood that SARS-CoV-2 can infect children as well as adults [46].

One study showed that during the early infection period, most patients had normal white blood cell counts; however, 56.8% of patients had leukopenia in cases of serious infection [46]. Patients with dyspnea were more frequently admitted to the intensive care unit (ICU) [46]. Most chest computed tomography (CT) showed bilateral patchy shadows or ground-glass opacity (GGO) in the lungs [5,6]. In another study, 86% of patients showed GGO by chest CT, and 29% of patients had consolidation [47]. Following the appearance of GGO, 75% of patient lung CTs showed reticular or interlobular septal thickening [48]. However, no direct cavitation, pleural effusion, lymphadenopathy or nodules were observed in the lungs of COVID-19 patients [49] (Table 1).

In view of the large amounts of cytokines produced during SARS-CoV infection, infection with SARS-CoV-2 similarly induces the production of proinflammatory cytokines such as, interleukin 1 beta (IL-1β), interferon gamma (IFN-γ), IP10 and monocyte chemoattractant protein 1 (MCP). Moreover, the levels of granulocyte colony stimulating factor (GCSF), IP10, MCP1, MIP1α and tumor necrosis factor- alpha (TNF-α) were found to be higher in Intensive care unit (ICU) than non-ICU patients [50]. However, secretion of immunosuppressive cytokines (e.g.,interleukin 4 [IL-4] and interleukin 10 [IL-10] by T-helper type 2 [Th2] cells was also increased during SARS-CoV-2 infection [6]) (Table 1).

The mortality rate of SARS-CoV-2-infected patients varies in different studies. A study of 41 patients showed a mortality rate of 15% [6], and the fatality of 138 patients in a clinical study was 4.3% [5]. However, Chen showed that the mortality was 3%, a number closer to the official national statistics of China (2.01%) [51]. These differences in mortality rates are possibly due to the difference in sample size. The reported mortality rate of SARS-CoV was 10%, and that of MERS-CoV was about 36% [11]. The currently estimated mortality rate of SARS-CoV-2 is therefore lower than that of SARS-CoV and MERS-CoV [51,52] (Table 1). In summary, SARS-CoV-2 spreads more rapidly but has a relatively lower fatality rate as compared with two other related coronaviruses.

## Diagnostic laboratory testing

### Hematology testing

The number of total leukocytes, lymphocytes and monocytes has been detected from hospitalized patients with COVID-19 [53]. Approximately 25% of cases formed leucopenia [6]. Moreover, lymphopenia was observed in 63–70.3% of patients [5,6]. CD4^+^ or CD8^+^ T-cell numbers decreased as the disease severity increased [6,54]. Patients with severe cases had more prominent abnormalities than those with non-severe cases.

## Nucleic acid testing

Virus from throat swabs, blood, urine, stool or respiratory tracts have been assessed by fluorescent reverse transcription-polymerase chain reaction (RT-PCR) methods [55]. Primers and probes targeting *RdRp/helicase (Hel)*, *E, S, N* and replicase *ORF 1a/b* genes were designed and tested for SARS-CoV-2 [55]. RT-PCR showed that primers of the *E* and *RdRp* genes demonstrated better sensitivity than that of the N gene, and the limit of detection (LOD) of *RdRp/Hel* and *N* gene was lower than that of S gene and RdRp-P2 [18]. Chan *et al.* showed that the COVID-19-RdRp/Hel assay (*RdRp/Hel* probe, FAM-TTAAGATGTGGTGCTTGCATACGTAGAC-lABkFQ) was significantly more sensitive than the RdRp-P2 assay for the detection of SARS-CoV-2 RNA, and detection targeted *ORF1a/b*, *ORF1b-nsp14*, *RdRp*, *S*, *E* or *N* genes was with less specificity for SARS-CoV-2 [18].

Moreover, a previous study showed that the specific probe *RdRP*_SARSr-P2 (FAM-CAGGTGGAACCTCATCAGGAGATGC-BBQ) detected only the SARS-CoV-2 RNA transcript but not the SARS-CoV RNA [55]. However, a novel RT-PCR assay showed that targeting *RdRp2* was a non-specific assay for SARS-CoV-2, because this detected other betacoronaviruses such as SARS-CoV [18]. The COVID-19-*RdRp/Hel* assay had the lowest LOD *in vitro* and higher sensitivity and specificity, which helped to reduce the false-negative rate and improve the laboratory diagnosis of COVID-19 [18].

SARS-CoV-2 viral load is detectable from throat- and lung-derived samples; however, blood and urine have not yet yielded virus [56]. Patients with COVID-19 produced the highest viral load near symptom presentation, which may be the reason for the fast spread of the virus [57]. An observational cohort showed that viral load in saliva was highest following symptom onset in the first week, then gradually declined with time [56]. Endotracheal aspirate viral load was available from day 8 after symptom onset and did not significantly decline thereafter [57]. Following symptom onset, virus load in 33% patients could be detected for 20 days or longer in that study. Viral load in respiratory tract specimens was about sixfold higher than that in the nonrespiratory tract specimens [57]. In addition, elder patients reportedly had a greater virus load than that of younger patients. Studies showed that higher initial viral load was related to the severity of COVID-19 symptoms [57].

Although the positive test ratio was only 47.4% in previous study, it was improved by novel assay methods [58]. The sample quality, collection time, detection kits and technical abilities of clinical doctors may affect the accuracy of detection. Therefore, precise diagnosis of COVID-19 should be combined with CT scans and nucleic acid testing.

## Viral genome sequencing

Bronchoalveolar lavage fluid or throat swabs from patients have been sequenced, and viral genomes were searched via BLAST with the SARS-CoV-2 sequence [38,39]. Genome sequencing has also been used to identify patients with suspected infection.

## Serology testing

In addition to hematological detection and nucleic acid testing, serological diagnosis is important for patients who present late with a very low viral load, below the detection limit of RT-PCR assays [59]. IgM and IgG titers were relatively increased on day 5 and rapidly raised 10 days after symptom onset in most patients [59,60]. The IgM-positive rate increased from 50 to 94%, whereas the IgG-positive rate increased from 81 to 100% [60]. In addition, the IgM- and IgG-positive rates were not significantly different before and after patients were found to be virally negative [61]. Therefore, virus-specific IgM and IgG serological testing may be used to confirm current or previous infection with SARS-CoV-2.

## Animal models for COVID-19

An ideal animal model for COVID-19 would reflect the clinical signs, viral replication and pathology displayed in humans. Non-human primate models (rhesus, cynomolgus macaques, African green monkeys, common marmoset, squirrel monkeys and mustached tamarins) have all been evaluated as models of SARS-CoV infection [62]. All non-human primates had pneumonia, cough and respiratory distress after virus infection [15]. Mice (BALB/c, C57BL6 and 129S strains) supported SARS-CoV replication and showed clinical signs of SARS [15]. *Rag1*^−/−^ mice, *CD1*^−/−^ mice, *STAT1*^−/−^ mice and Beige mice have been used to determine the role of immune effectors of the virus [63]. *AC70* and *AC63* transgene-positive mice also showed clinical manifestation after SARS-CoV infection, demonstrating their usefulness to study the pathogenesis and evaluation of vaccines and other therapeutics [63]. Hamsters also have been used to study immuno-prophylaxis and drug research as they harbored high viral titers and pulmonary histopathology upon virus infection [63]. Ferrets are another appropriate animal model to study this respiratory virus because the clinical symptoms, viral titers and histologic changes were similar to those of patients with virus infection [63]. ACE2, the receptor of SARS-CoV, was also identified as the functional receptor for SARS-CoV-2, therefore, mice, hamsters and ferrets may be animal models for studying the SARS-CoV-2 [63]. An article reported in *Science* shows that SARS-CoV-2 can replicate in the upper respiratory tract of ferrets, indicating that ferrets represent an ideal animal model for evaluating antiviral drugs or vaccine candidates against COVID-19 [64]. In addition, the domestic cat has shown multifocal pulmonary consolidation with infection of SARS-CoV, and SARS-CoV-2 can replicate efficiently in cats and transmit between cats via the airborne route [64]. By contrast, dogs, pigs, chickens and ducks are poorly susceptible to SARS-CoV-2 [64].

## Treatment for COVID-19

No specific therapeutic medicine has been approved for the treatment of SARS-CoV-2 infection. All patients are given empirical antibiotic or antiviral drugs. Moxifloxacin or levofloxacin are empirically used to treat early coinfections with bacteria [65]. Linezolid is effective against *Streptococcus pneumoniae* and *Staphylococcus aureus* and is combined with nemonoxacin in cases of severe infection [65].

A combination of lopinavir and ritonavir has been used to treat COVID-19 because the lopinavir/ritonavir (LPV/r) combination has been confirmed to be effective against SARS-CoV and MERS-CoV [6,66,67]. Another antiviral drug, remdesivir (RDV), was predicted to be efficacious against COVID-19 by target-based virtual ligand screening [68]. RDV is a novel nucleotide analog prodrug that is in development and has demonstrated effective pan-CoV therapy. The first case of SARS-CoV-2 infection in the USA was successfully treated with RDA. Moreover, a randomized, double-blind, parallel-controlled Phase III clinical trial was conducted to recruit SARS-CoV-2-infected patients [4,69].

Favipiravir is a nucleoside analog that can lead to lethal viral mutagenesis, chain termination or the inhibition of nucleotide biosynthesis [70]. Favipiravir in combination with oseltamivir, which was given to patients infected with SARS-CoV-2, has been used to treat severe influenza [45]. Oseltamivir, a neuraminidase inhibitor, is recommended as an antiviral treatment for influenza and has been widely used to inhibit COVID-19 in China [45,70]. Other neuraminidase inhibitors, zanamivir and peramivir, are also effective treatments for MERS-CoV [71]. At this time, these are all empirical therapies for COVID-19. However, whether oseltamivir or zanamivir are effective treatments for COVID-19 also needs further study.

Arbidol (ARB) was licensed for the treatment of influenza and other respiratory viral infections in Russia and China [72,73]. Blaising and coauthors consider ARB to be a broad spectrum antiviral drug [72]. In addition, ARB has been reported to inhibit SARS-CoV *in vitro*. Therefore, a clinical trial of ARB-treated COVID-19-positive patients has been registered [65].

Glucocorticoids have been commonly used in patients with SARS-CoV or MERS-CoV infection [74,75]. Glucocorticoids prolonged the survival time of SARS cases [75]. Patients with COVID-19 were given glucocorticoids in some hospitals in China. However, the mortality rate did not decrease with corticosteroid treatment in patients infected with SARS-CoV-2, and viral clearance was not delayed [6,74]. Therefore, it is still controversial whether corticosteroids should be used to treat SARS-CoV-2 infections [69].

Chloroquine/hydroxychloroquine was used as an antimalarial, broad spectrum antiviral drug, and has been broadly used in autoimmune diseases including lupus and rheumatoid arthritis [76]. A recent study indicates that chloroquine and the antiviral drug RDV-inhibited SARS-CoV-2 *in vitro* [77]. Clinical symptoms of patients treated with chloroquine were obviously relieved: for example, more rapid decline in fever and improvement of lung CT [77]. Chloroquine was suggested to treat COVID-19 in the SARS-CoV-2 treatment guidelines by the Chinese medical advisory. Chloroquine has been highly effective in reducing SARS-CoV-2 viral replication by increasing endosomal pH and interfering with the glycosylation of cellular receptors [76]. Moreover, chloroquine was probably the first molecule to be used to treat COVID-19.

Another antiviral drug, nelfinavir, an HIV protease inhibitor, was predicted to be a potential inhibitor of COVID-19 [26]. In addition, cytokine immunotherapy with IFN-α, a broad spectrum antiviral drug, has been used to inhibit HBV. IFN-α was used to treat patients infected with SARS-CoV-2 according to established guidelines [70]. IFN-α combined with LPV/r was shown to be beneficial for treatment of COVID-19 [78].

Tocilizumab is a humanized anti-IL-6-receptor (IL-6R) monoclonal antibody that inhibits IL-6 signaling and is used as a treatment in rheumatoid arthritis [79]. IL-6 is one of the most important cytokines involved in COVID-19-induced cytokine storms. Tocilizumab (TCZ) has been used to treat COVID-19 in China and Italy. TCZ was recommended for COVID-19 patients to prevent or treat cytokine storms and could reduce the mortality of COVID-19 [80].

Other drug types such as RNA synthesis inhibitors (TDF and 3TC) and an FP (EK1), have also effectively inhibited SARS-CoV-2 *in vitro*. In addition, traditional Chinese medicines (Lian Hua Qing Wen Capsules, Shu Feng Jie Du Capsules) have also been used to treat COVID-19 in the latest version of the Diagnosis and Treatment of pneumonia induced by COVID-19 [81]. In addition, human seroalbumin and γ-immunoglobulin were given to some patients with severe infections [82]. In conclusion, there are specific vaccines or antiviral drugs for COVID-19. All of the drugs described above have shown some usefulness in treating SARS-CoV-2 infections, and their efficacy merits further study.

## Conclusion & prospects

**Figure 3.**
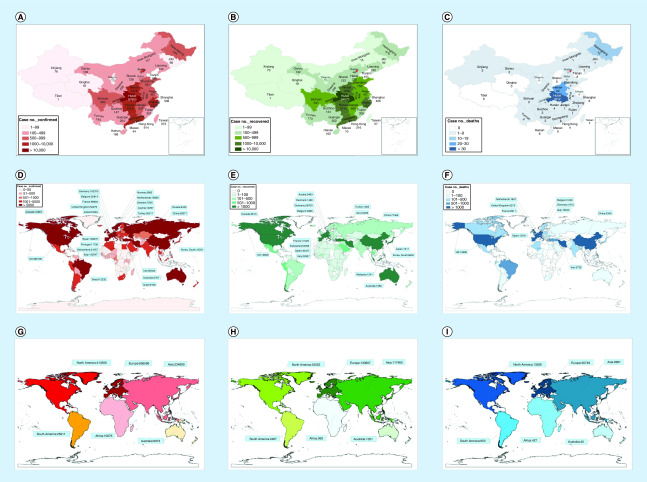
Distribution of laboratory-confirmed cases, cured cases and death cases of 2019 coronavirus disease (COVID-19). Distribution of laboratory-confirmed cases, cured cases and death cases of 2019 coronavirus disease (COVID-19 **(A, B & C)** in China by province/region as of 7 April 2020. **(D, E & F)** Distribution of laboratory-confirmed cases, cured cases and death cases of 2019 coronavirus disease (COVID-19) globally by country as of 7 April 2020. The number of confirmed cases ≥5000 by countries was labeled in **(D).** All cured cases and death cases by countries were labeled in **(E & F)**. **(G, H & I)** Distribution of laboratory-confirmed cases, cured cases and death cases of COVID-19 in the word by continent as of 7 April 2020. Up to 7 April 2020, the number of infected countries was at least 184. The number death patients are at least 74,596 and patients have been cured 277,420 in all the word. All the data are from Johns Hopkins resource center and National Health Commission of Chinese.

COVID-19 is a serious human infectious disease of global concern. As of 7 April 2020, SARS-CoV-2 has infected a total of 134,784 patients globally at least (Figure 3D & G). The COVID-19 outbreak poses a serious challenge to China and the whole world; it has profoundly affected public health. Although the overall mortality rate of SARS-CoV-2 appears to be lower than that of SARS-CoV and MERS-CoV (Table 1), the transmissibility of COVID-19 is more rapid. Moreover, the fatality rate of elderly patients with reduced immunity or chronic diseases is as high as 15% [6]. In addition, asymptomatic carriers may be a potential source of infection, sustaining a local epidemic and global spread [83]. Therefore, COVID-19 may cause disruptions to the global public health system for an extended period of time.

The outbreak of COVID-19 has shown that there are still shortcomings in the prevention of public health diseases such as the lack of awareness of the frontline doctors and the early vigilance and attention from the government. Meanwhile, the awareness of the general public to health concerns also must be improved. Individuals should develop good living habits: for example, keeping away from wild animals, not consuming wild animals, maintaining hand hygiene – among others. In addition, the development of targeted antiviral drugs should be anticipated and accelerated in the future.

Certainly, vigorous measures to prevent contagion have been taken in China and other countries. To prevent the spread of infection, the Chinese government imposed a full lockdown and canceled public events such as the new year festival; contact with wild animals was also restricted, and travel was reduced with screening at airports, railway stations and subway stations [84]. Moreover, two emergency hospitals in Wuhan were constructed, and army medical units and medical staff from other areas were deployed to help prevent infections in Wuhan [84]. The WHO and various governments advised people to reduce public activity, maintain social distance, wear masks, wash their hands frequently, and practice respiratory hygiene. As of 7 April 2020, the number of fully recovered patients was 277,420 worldwide (Figure 3E & H), and the number of recovered patients was 77,468 in China (Figure 3B). Therefore, we are confident that the outbreak of COVID-19 will be effectively curbed.

Most countries have isolated any suspected cases as rapidly as possible to contain infection and prevent local outbreaks. The ability to rapidly test patients suspected of having a SARS-CoV-2 infection is the cornerstone of case isolation. The experience gained from SARS-CoV and MERS-CoV by the health community over the last 20 years could also help in dealing with SARS-CoV-2 infections throughout the world.

## Future perspective

To date, no SARS-CoV-2-specific antiviral drugs or vaccines have been described for COVID-19. Therefore, a safe and stable vaccine for COVID-19 is urgently needed, and it is expected to be ready within 18 months [81]. Vaccines specific for SARS-CoV-2 will immunize people worldwide in the future. It is hoped that drugs specifically targeted for COVID-19 also will be widely developed. Meanwhile, the origin, intermediate host, structure and pathogenesis of SARS-CoV-2 will be the focus of future research. Moreover, prediction of whether the similar coronaviruses can infect humans will be import for all the world. We hope that vaccines specific for the similar coronaviruses will be developed in advance to prevent epidemics in the world.

**Executive summary**

- Coronavirus disease 2019 (COVID-19) was first reported in China and currently poses a serious challenge worldwide. The pneumonia was caused by a novel coronavirus named SARS-CoV-2.
- More than 1,347,804 cases of COVID-19 and 74,596 deaths have been reported as of April 7, 2020 according to data from Johns Hopkins Resource Center.
- SARS-CoV-2 belongs to *Betacoronavirus*, a large genus of viruses prevalent in nature. SARS-CoV-2 has 82% nucleotide identity with human SARS-CoV and 96% nucleotide identity with bat SARS coronavirus (SARSr-CoV-RaTG13).
- The fatality rate of COVID-19 has been approximately 3.4% and the R0 has ranged from 1.4 to 6.4. Compared with previous coronavirus outbreaks, COVID-19 has been reported to have a lower mortality rate and more rapid transmissibility and caused severe acute respiratory syndrome similarly to SARS.
- The main symptoms are fever, cough, shortness of breath, leukopenia and pneumonia.
- Diagnostic laboratory testing of COVID-19 includes hematology testing, nucleic acid testing, viral genome sequencing and serology testing. To date, no specific antiviral drugs or vaccines for COVID-19 have been developed. Therefore, empirical antiviral drugs (lopinavir/ritonavir, favipiravir, oseltamivir, zanamivir and peramivir, arbidol), antibiotic drugs (moxifloxacin, levofloxacin, linezolid), chloroquine/hydroxychloroquine, glucocorticoids, monoclonal anti-inflammatory antibody (tocilizumab), have been used to treat SARS-CoV-2 infection. Traditional Chinese medicines are also used for therapy during infection with SARS-CoV-2.
- This review is presented in the hope of helping the public effectively recognize and combat COVID-19 and to provide a reference for future studies or outbreaks.
